# Highly Sensitive Luminescent Bioassay Using Recombinant *Escherichia coli* Biosensor for Rapid Detection of Low Cr(VI) Concentration in Environmental Water

**DOI:** 10.3390/bios11100357

**Published:** 2021-09-27

**Authors:** Guey-Horng Wang, Chiu-Yu Cheng, Teh-Hua Tsai, Pin-Kuan Chiang, Ying-Chien Chung

**Affiliations:** 1 Research Center of Natural Cosmeceuticals Engineering, Xiamen Medical College, Xiamen 361008, China; 201400080001@xmmc.edu.cn; 2 Department of Biological Science and Technology, China University of Science and Technology, Taipei 115, Taiwan; cycheng@cc.cust.edu.tw (C.-Y.C.); pkchiang2021@gmail.com (P.-K.C.); 3 Department of Chemical Engineering and Biotechnology, National Taipei University of Technology, Taipei 10608, Taiwan; thtsai@ntut.edu.tw

**Keywords:** biosensor, hexavalent chromium, limit of detection, reporter gene

## Abstract

In this study, we constructed a recombinant *Escherichia coli* strain with different promoters inserted between the chromate-sensing regulator *chrB* and the reporter gene *luxAB* to sense low hexavalent chromium (Cr(VI)) concentrations (<0.05 mg/L); subsequently, its biosensor characteristics (sensitivity, selectivity, and specificity) for measuring Cr(VI) in various water bodies were evaluated. The luminescence intensity of each biosensor depended on pH, temperature, detection time, coexisting carbon source, coexisting ion, Cr(VI) oxyanion form, Cr(VI) concentration, cell type, and type of medium. Recombinant *lux*-expressing *E. coli* with the T7 promoter (T7-lux-*E. coli*, limit of detection (LOD) = 0.0005 mg/L) had the highest luminescence intensity or was the most sensitive for Cr(VI) detection, followed by *E. coli* with the T3 promoter (T3-lu*x-E. coli*, LOD = 0.001 mg/L) and that with the SP6 promoter (SP6-lux-*E. coli*, LOD = 0.005 mg/L). All biosensors could be used to determine whether the Cr(VI) standard was met in terms of water quality, even when using thawing frozen cells as biosensors after 90-day cryogenic storage. The SP6-lux-*E. coli* biosensor had the shortest detection time (0.5 h) and the highest adaptability to environmental interference. The T7-lux-*E. coli* biosensor—with the optimal LOD, a wide measurement range (0.0005–0.5 mg/L), and low deviation (−5.0–7.9%) in detecting Cr(VI) from industrial effluents, domestic effluents, and surface water—is an efficient Cr(VI) biosensor. This unprecedented study is to evaluate recombinant lux *E. coli* with dissimilar promoters for their possible practice in Cr(VI) measurement in water bodies, and the biosensor performance is clearly superior to that of past systems in terms of detection time, LOD, and detection deviation for real water samples.

## 1. Introduction

Hexavalent chromium (Cr(VI)) is widely used in metal refining, leather tanning, wood preservation, and chemical and refractory processing; it is also applied to produce stainless steel, textile dyes, mimeographs, and plastics and as an anticorrosion agent [1,2]. If wastewater containing Cr(VI) is not treated properly, it may directly or indirectly pollute the water body or soil. Cr(VI) has been listed as a hazardous substance by the Agency for Toxic Substances and Disease Registry since 2011. Cr(VI) can damage DNA and cause varied toxicity, mutagenicity, and carcinogenicity, because it can quickly pass through the cell membrane and enter the cell given its high solubility, bioavailability, and mobility [3,4,5]. In Taiwan, environmental protection agencies have set 0.5 mg/L as the maximum allowable level (MAL) for Cr(VI) in industrial and domestic effluents and 0.05 mg/L as the MAL in surface water and fishery water. Thus, the need for sensitive Cr(VI) detection is high, but the design of such sensors is challenging.

Conventional analytical techniques such as atomic absorption spectroscopy, inductively coupled mass spectrometry, and ultraviolet–visible spectrophotometry are sensitive and reliable for Cr(VI) detection but are expensive, laboratory-bound, and time-consuming [6]. Additionally, they only measure total metals but cannot distinguish between bioavailable and biounavailable metal concentrations or analyze the toxicity of metals [7,8]. By contrast, biological methods are useful alternatives because they can detect bioavailability and are low cost, easy to use, small, portable, highly sensitive, and highly selective [5,9]. Of the biological methods, bacterial biosensors are suitable for application as environmental sensors and early warning devices, even for on-field measurements [10].

Some Cr(VI) measurement techniques based on biological reactions have been developed. The performance of enzyme-based biosensors (e.g., amperometric enzyme and urease) for Cr(VI) detection is easily affected by the environment [11,12]. V79 cell biosensors have a wide Cr(VI) measurement range (0.97–19.4 mg/L), but their cost is high compared with that of microbe biosensors [13]. The performance of microbial-based biosensors (e.g., sulfur-oxidizing bacteria and *Enterobacter aerogenes* T2) for Cr(VI) detection is easily affected by coexisting metal ions, and these biosensors cannot measure Cr(VI) concentrations ≤0.05 mg/L [14,15]. The limit of detection (LOD) of microbial fuel cell (MFC)-based biosensors (inoculated with *Exiguobacterium aestuarii* YC211) is 2.5 or 5 mg/L depending on the system configuration [16,17]; however, the LOD is insufficiently sensitive to detect low Cr(VI) concentrations (e.g., ≤0.05 mg/L).

To detect low Cr(VI) concentrations, an engineered biosensor (whole-cell biosensor) should be considered. Many strains that can remove Cr(VI) have been discovered, such as *Pseudomonas* spp., *Streptococcus lactis*, *Stenotrophomonas maltophilia*, *Pannonibacter phragmitetus*, *Cupriavidus metallidurans*, and *Ochrobactrum* sp., and a key gene—the chr gene (responsible for Cr(VI) reduction)—has been identified [18,19,20,21]. However, considerable differences exist in the sequences of chr genes from different strains, which results in disparate reduction activities [21]. Among such strains, the genus *Ochrobactrum* has high Cr(VI) reduction ability; thus, the *chr* gene from *Ochrobactrum* spp. should be used to measure Cr(VI) [19,22].

A biosensor can be genetically engineered by placing a reporter gene, such as *lacZ*, *gfp*, *luc*, or *lux*, under the control of a transcriptional activator [23]. Of these reporter genes, *lux* and *luc* can rapidly obtain detectable signals, whereas *gfp* requires a long induction period for obtaining environmental responses [10], and the LOD of the biosensor using *gfp* as the reporter gene is often inferior to that of the biosensor using *lux* or *luc* as the reporter gene [24]. Thus, using the reporter gene from luminescent bacteria to construct a recombinant bacterium has considerable application potential for achieving satisfactory LOD. To increase the sensitivity, signal intensity, and response speed of whole-cell biosensors, a suitable promoter should be selected. The common promoters SP6, T3, and T7 have similar but distinct specificities [25]. T7 is a strong promoter that actuates gene expression tuned to the highest level, thus amplifying the detection signal and producing a high LOD [26]. By contrast, relatively weak promoters (SP6 and T3) may adapt to environmental variation, which produces different signal characteristics and distinct detection ranges [27]. Thus, the LOD and measurement range of the biosensor would be improved or expanded with the insertion of suitable promoters into recombinant luminescent bacteria for moderate control of the expression of reporter genes [28,29].

Recombinant luminescent bacterial biosensors have been constructed to detect Hg^2+^, Cu^2+^, Pb^2+^, Ag^+^, Cd^2+^, Ni^2+^, Zn^2+^, As^3+^, and As^5+^ [7,30]. However, few recombinant biosensors have been developed for Cr(VI) detection and for measuring Cr(VI) in real water samples. Smutok et al. (2011) constructed *Hansenula polymorpha* recombinant cells to detect Cr(VI), but the LOD (0.52 mg/L) was poor [31]. Branco et al. (2013) constructed two whole-cell biosensors, namely pCHRGFP1 *Escherichia coli* and pCHRGFP2 *O. tritici*, including the *chrB* regulator gene and *gfp* reporter gene to detect bioavailable Cr(VI). The recombinant *E. coli* biosensor was more sensitive than the recombinant *O. tritici* biosensor, and its LOD was 0.0194 mg/L in the 3-h detection time; however, complete and detailed evaluation in real water samples was lacking. To detect ≤0.05 mg/L Cr(VI) (MAL as per regulations), a strong promoter (e.g., T7 promoter) fused with a sensitive reporter gene (e.g., *luxAB* gene) should be used to shorten the detection time, enhance the luminescence intensity, and improve the LOD.

In this study, recombinant *E. coli* strains were engineered to carry the *chrB* plasmid from *O. anthropi* YC211 and contain various promoters (T7, T3, or SP6) to regulate *luxAB* expression. After the optimization of the promoter and the regulation of the *luxAB* expression level in *E. coli*, the recombinant luminescent biosensors could identify bioavailable Cr(VI) in real water samples of various source.

## 2. Materials and Methods

### 2.1. Bacterial Strains, Gene Cloning, and Biosensor Construction

**Figure 1:**
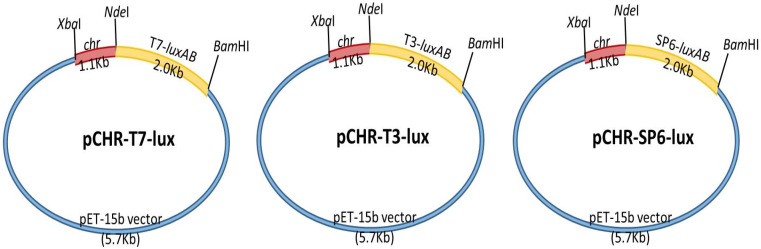
Construction of pCHR-T7-luxAB, pCHR-T3-luxAB, and pCHR-SP6-luxAB plasmids.

To clone *chrB* in *O. anthropi* YC211, this gene was amplified using the primer set XbaI-chrf and NdeI-chrr through polymerase chain reaction (PCR). To clone T3-*luxAB*, T7-*luxAB*, or SP6-*luxAB*, *luxAB* in *Vibrio fischeri* was amplified using the primer set *Nde*I-T7-luxABf, *Nde*I-T3-luxABf, or *Nde*I-SP6-luxABf (lux-forward primer) and *Bam*HI-luxABr (lux-reverse primer) through PCR [32]. The sequences of the primers are provided in Table 1. The resultant DNA fragments were all introduced into the pET-15b vector plasmid (Promega, Madison, WI, USA). The recombinant plasmids were entitled pT7-*luxAB*, pT3-*luxAB*, pSP6-*luxAB*, and pCHR. In short, the plasmids were then transferred to *E. coli* BL21; these cells were cultivated on Luria–Bertani (LB) agar plates. Eventually, isolated pT7-luxAB, pT3-luxAB, pSP6-luxAB, and pCHR plasmids were cut using NdeI/BamHI and XbaI/NdeI [10]. Next, pCHR was ligated to pT7-luxAB, pT3-luxAB, and pSP6-lux fragments, respectively, by using T4 DNA ligase (New England BioLabs, Beverly, MA, USA) and pCHR-T7-luxAB, pCHR-T3-luxAB, and pCHR-SP6-luxAB were constructed. The resulting plasmids were introduced into the pET-15b vector plasmid. Subsequently, the plasmids were transformed into *E. coli* BL21 to produce the corresponding chromate biosensors. The restriction enzymes were obtained from New England BioLabs (USA). Vector DNA was obtained using the QIAEX II gel extraction kit (Qiagen, Hilden, Germany). Figure 1 demonstrates the construction of the three recombinant plasmids.

### 2.2. Bacterial Growth

*E. coli* with pCHR-T7-luxAB (T7-lux-*E. coli*), pCHR-T3-luxAB (T3-lux-*E. coli*), and pCHR-SP6-luxAB (T3-lux-*E. coli*) (all initial concentrations of 10^7^ cfu/mL) were cultivated in LB broth containing a final ampicillin concentration of 50 mg/L, and Cr(VI) concentration of 0.05 mg/L at 37 °C at 200 rpm on an orbital shaker. After 18 h, cultures were diluted 100-fold into modified Tris minimal salts medium (TMM) containing 6.06 g/L Tris, 4.68 g/L NaCl, 1.49 g/L KCl, 1.07 g/L NH_4_Cl, 0.43 g/L Na_2_SO_4_, 0.2 g/L MgCl_2_.6H_2_O, 0.03 g/L CaCl_2_, 0.23 g/L Na_2_HPO_4_, 50 mg/L ampicillin, and 0.3% glucose, as per the previously described method of Mergeay et al. (1985) but with a slight modification [33]. In this study, *E. coli* was used as a control. To evaluate the feasibility of using frozen cells instead of fresh cells, the overnight culture grown in modified TMM was prepared as bacterial glycerol stocks and stored at −80 °C. To evaluate the relationship between bacterial growth and the luminescence intensity of recombinant *E. coli*, the cultures were incubated at 37 °C at 200 rpm on an orbital shaker, and optical density (OD) at 600 nm and the luminescence intensity were measured at specific intervals. Sampling 200 μL of the culture to a 96-well microplate, and then placing it under a microplate luminometer (Titertek-Berthold, Pforzheim, Germany), the luminescence intensity (expressed as relative light units (RLU)) was measured. All chemicals utilized in the experiment were analytical grade.

### 2.3. Determination of Optimal Conditions

After 18 h of cultivation in modified TMM, 1 mL of culture containing 10^8^ cfu/mL T7-lux-*E. coli*, T3-lux-*E. coli*, or SP6-lux-*E. coli* was inoculated into 100 mL of modified TMM with 0.5 mg/L Cr(VI) and incubated at 37 °C for 5 h. Cr(VI) was added as the oxyanion form of CrO_4_^2−^, unless otherwise stated. The luminescence intensity was measured every 20 min. The effects of temperature (15 °C–45 °C) and pH value (4–9) on the luminescence intensities of the three recombinant *E. coli* strains were evaluated separately. Temperature was controlled using a constant-temperature incubator, and pH was adjusted using 1 N HCl or NaOH solution. To assess the effects of coexisting carbon sources on the luminescence intensities of the three recombinant *E. coli* strains, formic acid, acetic acid, citric acid, acetone, or fructose was added to the modified TMM, respectively. The final concentrations of these carbon compounds in the medium were 300 μM. To evaluate the effects of Cr(VI) oxyanion forms on the luminescence intensities of the three recombinant *E. coli* strains, CrO_4_^2−^ or Cr_2_O_7_^2−^ was introduced to the modified TMM.

The Cr(VI) concentrations added were 0.05, 0.5, and 5 mg/L. Coexisting cations (Ni(II), Co(II), Cd(II), Zn(II), Cu(II), and Cr(III)) and coexisting anions/similar structural configurations of CrO_4_^2−^ (SO_4_^2−^, PO_4_^3−^, and AsO_4_^2−^) were added to modified TMM to evaluate their effects on the luminescence intensities of the three recombinant *E. coli* strains. The concentrations of coexisting ions were 0.5–5 mg/L, and the Cr(VI) concentrations evaluated were 0.02, 0.5, and 5 mg/L. To evaluate the performance of frozen cells for detecting 0.05 and 5 mg/L Cr(VI), frozen cells after 30-, 60-, 90-, and 120-day storage were examined. The luminescence emitted by T7-lux-*E. coli*, T3-lux-*E. coli*, and SP6-lux-*E. coli* was stable and the highest was observed at 1.5–2.0 h, 1–1.5 h, and 0.5–1.0 h after incubation, respectively. Therefore, after 1.5-h incubation for T7-lux-*E. coli* cells, 1-h incubation for T3-lux-*E. coli* cells, and 0.5-h incubation for SP6-lux-*E. coli* cells, 200 μL of the cultures was sampled, and the luminescence intensity of these biosensors was measured immediately. After the completion of incubation, luminescence intensity was measured every 5 min over 20 min, and the five measured values were then averaged. The effects (luminescence intensity and stability time) of medium types (LB and modified TMM) and the Cr(VI) concentration (0.01–10 mg/L Cr(VI)) on luminescence characteristics of the three recombinant *E. coli* strains were evaluated. The cultures were incubated at 37 °C for 5 h, and the luminescence intensity was periodically measured. Incubation temperature, pH, and Cr(VI) concentration were controlled at 37 °C, pH 7, and 0.5 mg/L, respectively, unless otherwise stated. Measurements from at least three independent experiments were obtained, each performed at least in triplicate.

### 2.4. Establishment of Calibration Curve and Measurement of Real Water Samples

To establish the relationships between the Cr(VI) concentration and the luminescence intensity of the three recombinant *E. coli* biosensors, we mixed 2 mL deionized water containing Cr(VI) at different concentrations and 2 mL culture containing recombinant luminescent *E. coli* cells (final concentration after mixing: 5 × 10^6^ cfu/mL). The culture was prepared using the thawed cells after 90-day cryogenic storage. The optimal incubation time and conditions were according to the results in previous experiments. Calibration curves were plotted on the basis of the linear regression of the luminescence intensity at each corresponding Cr(VI) concentration. That region (point) of the calibration, where there is a significant change in sensitivity (i.e., a break in the slope of the calibration), was defined as LOD concentration.

To verify that the established curves and methods were valid and feasible, similar solutions were prepared as mentioned in the earlier text, but industrial effluents (from Guishan Industrial Sewage Treatment Plant, Taoyuan City, Taiwan) and domestic effluents (from Dihua Sewage Treatment Plant, Taipei City, Taiwan) and surface water (from Tamsui River, New Taipei City, Taiwan) were used instead of pure Cr(VI) solution. Considering practical application, the performance of recombinant *E. coli* cells was examined after cryogenic storage. Thus, thawed cells after 90-day cryogenic storage were used instead of fresh cells for Cr(VI) measurement in real water samples. The Cr(VI) concentration in the real water samples was separately measured using the colorimetric 1,5-diphenylcarbazide (DPC) method [34] and the developed recombinant *E. coli* biosensors. Experimental data were collected from at least three independent experiments.

## 3. Results and Discussion

### 3.1. Time-Dependent Induction of Three Recombinant E. coli Biosensors with Cr(VI)

**Figure 2:**
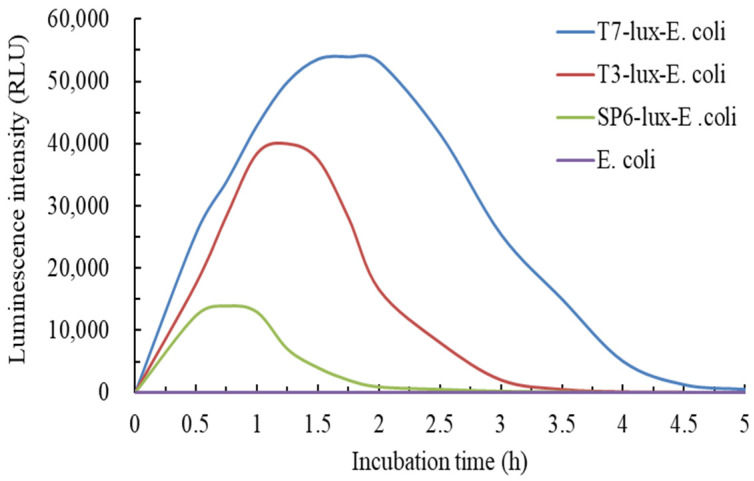
Time-dependent induction of luminescence from the developed three recombinant luminescent *E. coli* biosensors and *E. coli* was used as a control. (initial cell concentration: 10^6^ cfu/mL, culture media: modified TMM with 0.5 mg/L Cr(VI), culture temperature: 37 °C, stirring speed: 200 rpm).

In the preliminary experiment, all the logarithmic growth phases of three recombinant *E. coli* cells were occurred at 8–20 h incubation. During this period, the luminescence intensities of the three recombinant *E. coli* strains were proportional to the bacterial growth. Therefore, we set the inoculation time of the three recombinant *E. coli* strains at 18 h after incubation in the subsequent experiment. Figure 2 indicated a comparison of the time-dependent induction of luminescence from the three recombinant *E. coli* biosensors (i.e., T7-lux-*E. coli*, T3-lux-*E. coli*, and SP6-lux-*E. coli*) with 0.5 mg/L Cr(VI). The results indicated that the luminescence intensity of three recombinant *E. coli* biosensors rapidly increased, plateaued at 0.5 h, and then decreased during incubation. The luminescence intensity emitted from *E. coli* (control) maintained zero. However, this intensity variation was different from the luminescence caused by the *gfp* reporter gene, which slowly increased and plateaued [10]. This disparity was potentially due to the biochemical nature of the reporter gene *luxAB* [35].

The results demonstrated that luminescence was stable and the highest at 1.5–2.0 h, 1–1.5 h, and 0.5–1.0 h after incubation for T7-lux-*E. coli*, T3-lux-*E. coli*, and SP6-lux-*E. coli*, respectively. The Cr(VI) detection time was shorter than that reported previously for *gfp*-based recombinant *E. coli* (3–5 h), V79 (6 h), *gfp*-based recombinant *O. tritici* (5 h) biosensors, and *luxCDABE*-based recombinant *Acinetobacter baylyi* ADPWH-recA (4.5–6 h) [5,10,13,36]. The maximum average luminescence intensities of T7-lux-*E. coli*, T3-lux-*E. coli*, and SP6-lux-*E. coli* cells were 53,633 ± 124.7, 37,966 ± 448.7, and 12,767 ± 205.5 RLU, respectively. The decrease in signal intensity of luminescence was shown as follows: T7-lux-*E. coli*, T3-lux-*E. coli*, and then SP6-lux-*E. coli*. SP6-lux-*E. coli* cells had the shortest stable period (0.5–1.0 h) for luminescence induction, but the intensity for T7-lux-*E. coli* cells was 4.2 times (53,633/12,767) higher than that of SP6-lux-*E. coli* cells. de Las Heras et al. (2012) adopted a similar approach to detect aromatic compounds by fusing the T7 promoter to control the expression of the *lux* operon to significantly increase the luminescence intensity [37].

### 3.2. Effects of Culture Conditions on Luminescence Intensity

**Figure 3:**
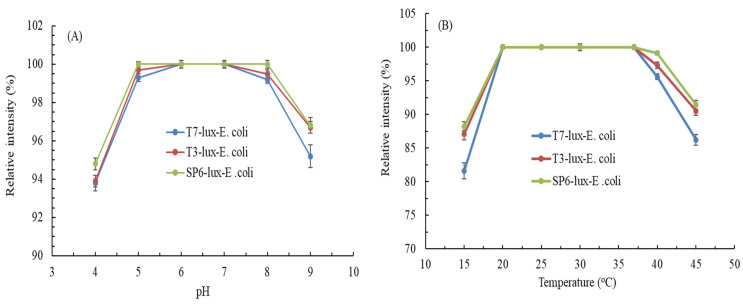
(**A**) Effects of pH on luminescence intensities of three recombinant *E. coli* biosensors induced with 0.5 mg/L Cr(VI). (**B**) Effects of incubation temperature on luminescence intensities of three recombinant *E. coli* biosensors induced with 0.5 mg/L Cr(VI) for 1.5 h (T7-lux-*E. coli*), 1 h (T3-lux-*E. coli*), and 0.5 h (SP6-lux-*E. coli*).

The effects of pH and incubation temperature on the luminescence intensities of the biosensors for Cr(VI) detection were evaluated (considering practical aspects). Relative intensities were calculated using luminescence intensities at pH 7 (pH effect study) and 37 °C (temperature effect study). Figure 3A illustrates the effects of pH on the luminescence intensity induced with 0.5 mg/L Cr(VI) for T7-lux-*E. coli*, T3-lux-*E. coli*, and SP6-lux-*E. coli* biosensors. The results demonstrated that the optimal pH range for the luminescence intensities of T7-lux-*E. coli*, T3-lux-*E. coli*, and SP6-lux-*E. coli* biosensors was 6–7, 5–7, and 5–8, respectively, with nonsignificant differences (*p* > 0.05). The SP6-lux-*E. coli* biosensor exhibited the highest pH adaptability among the biosensors. The T7-lux-*E. coli* biosensor was relatively unstable under pH change; nevertheless, the relative intensity of T7-lux-*E. coli* cells at pH 4 and 9 remained high at >93% (93.8% ± 0.41% and 95.2% ± 0.6%, respectively) compared with that at pH 7.

Figure 3B displays the effects of incubation temperature on the luminescence intensities induced with 0.5 mg/L Cr(VI) for T7-lux-*E. coli*, T3-lux-*E. coli*, and SP6-lux-*E. coli* biosensors. The results indicated that the optimal temperature range for the luminescence intensities of T7-lux-*E. coli*, T3-lux-*E. coli*, and SP6-lux-*E. coli* biosensors was 20–37 °C, 20–37 °C, and 20–40 °C, with nonsignificant differences (*p* > 0.05). The SP6-lux-*E. coli* biosensor exhibited the highest temperature adaptability, which may be due to the short incubation time at the set temperature. The incubation temperature had considerable effects on T7-lux-*E. coli* cells. At the temperature of 15 °C or 45 °C, the relative intensity of T7-lux-*E. coli* cells decreased to 81.6% ± 1.2% and 86.2% ± 0.82%, respectively. This inconsistent result was presumed to be related to the promoter (T3, T7, SP6) structure and composition, which determine the strength of promoter–target DNA bonds and adaptability to environment changes [38]. However, the pattern of the incubation periods (T7-lux-*E. coli* > T3-lux-*E. coli* > SP6-lux-*E. coli*) was another possible factor.

The effects of medium types (LB and modified TMM) and different Cr(VI) concentrations on the luminescence intensity and incubation time for the three recombinant *E. coli* biosensors were evaluated. The results indicated that the effects of medium types on the stability time for the three recombinant *E. coli* biosensors were not obvious when Cr(VI) at 0.01–5 mg/L was used. The optimal time of luminescence induction was still maintained at 1.5 h, 1 h, and 0.5 h for T7-lux-*E. coli*, T3-lux-*E. coli*, and SP6-lux-*E. coli* biosensors, respectively; however, the luminescence intensities of the biosensors in modified TMM decreased by 9.2%, 7.5%, and 4.6% for T7-lux-*E. coli*, T3-lux-*E. coli*, and SP6-lux-*E. coli* biosensors, respectively, in comparison with that in LB as medium. In LB containing the high Cr(VI) concentration of 10 mg/L, the optimal induction time for all biosensors was postponed to 2–2.5 h. However, modified TMM containing 10 mg/L Cr(VI) did not affect the optimal induction time. This delay in the induction time may be attributed to the LB composition being more complex and nutritious than modified TMM [10]. In conclusion, modified TMM is a suitable medium for biosensor application.

### 3.3. Effects of Coexisting Carbon Source, Cr(VI) Oxyanion Form, and Coexisting Ion on Luminescence Intensity

**Figure 4:**
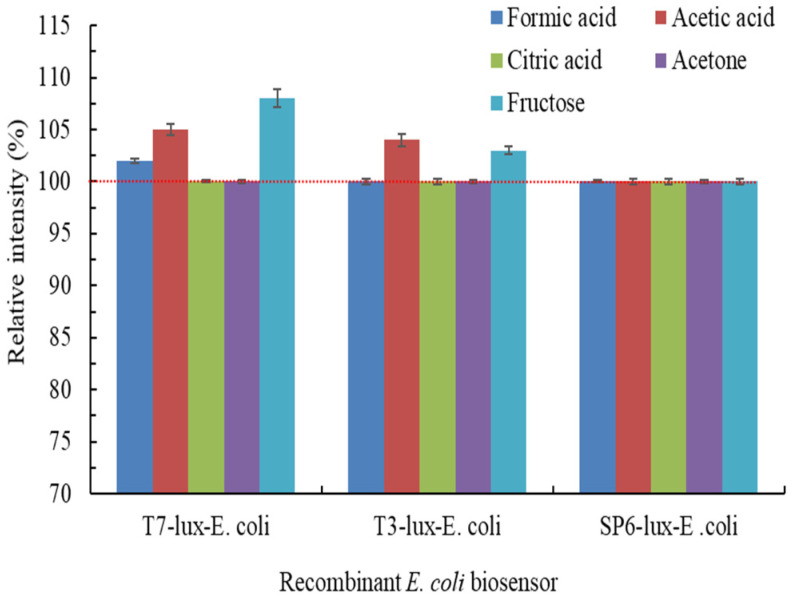
Effects of coexisting carbon sources at 300 μM on luminescence intensities of three recombinant *E. coli* biosensors induced with 0.5 mg/L Cr(VI) for 1.5 h (T7-lux-*E. coli*), 1 h (T3-lux-*E. coli*), and 0.5 h (SP6-lux-*E. coli*).

Figure 4 illustrates the effects of coexisting carbon sources at 300 μM on the luminescence intensities of the three recombinant *E. coli* biosensors. The different coexisting carbon sources may potentially improve or weaken the luminescence intensity; thus, the results may deviate considerably relative to the theoretically expected effects using modified TMM alone [10]. The results demonstrated that the coexistence of fructose or acetic acid with modified TMM induced a high luminescence intensity in the T7-lux-*E. coli* biosensor compared with modified TMM alone. Fructose or acetic acid synergistically increased luminescence by 8.1% ± 0.84% or 5.2% ± 0.51%, respectively. Only the coexistence of acetic acid with modified TMM induced a high luminescence intensity (4.2% ± 0.56%) in the T3-lux-*E. coli* biosensor compared with modified TMM alone. However, all coexisting carbon sources had negligible effects (*p* > 0.05) on Cr(VI) detection by the SP6-lux-*E. coli* biosensor, and this may be because the genetic assembly of the SP6-lux-*E. coli* biosensor is relatively less susceptible to environmental interference [27]. The elevated luminescence intensity may be attributed to be the easy biodegradation of fructose and acetic acid, which improved the related physiological activity of recombinant *E. coli* [39].

The Cr(VI) oxyanion form as CrO_4_^2−^ or Cr_2_O_7_^2−^ at 0.05–0.5 mg/L had nonsignificant effects (*p* > 0.05) on Cr(VI) detection by the three recombinant *E. coli* biosensors (relative intensity at 100–102.8%). However, the Cr(VI) form as Cr_2_O_7_^=^ at 5 mg/L caused a slight increase of 6.2% and 3.4% in the luminescence intensities of the T7-lux-*E. coli* and T3-lux-*E. coli* biosensors compared with that induced by the CrO_4_^=^ form, respectively. The Cr(VI) oxyanion form at the wide concentration range of 0.05–5 mg/L had negligible effects (*p* > 0.05) on Cr(VI) detection by the SP6-lux-*E. coli* biosensor. The coexisting ions of Ni(II), Co(II), Cd(II), Zn(II), Cu(II), Cr(III), SO_4_^2−^, PO_4_^3−^, and AsO_4_^2^^−^ (at concentrations of >3.5 mg/L) exerted significant effects (*p* < 0.05) on the luminescence intensities of the T7-lux-*E. coli* and T3-lux-*E. coli* biosensors compared with the control. However, these coexisting ions (0.5–5 mg/L) did not induce significant changes in Cr(VI) measurement by the SP6-lux-*E. coli* biosensor. Similar results were observed for *gfp*-based recombinant *E. coli* cells cultured with coexisting Cd(II), Zn(II), Co(II), Ni(II), Cu(II), SO_4_^2−^, PO_4_^3−^, or AsO_4_^2−^ [10]. Taken together, these results clearly illustrate that our recombinant luminescent biosensors possess favorable environmental adaptability, selectivity, and specificity under specific conditions.

### 3.4. Effects of Thawing Time on Luminescence Intensity

**Figure 5:**
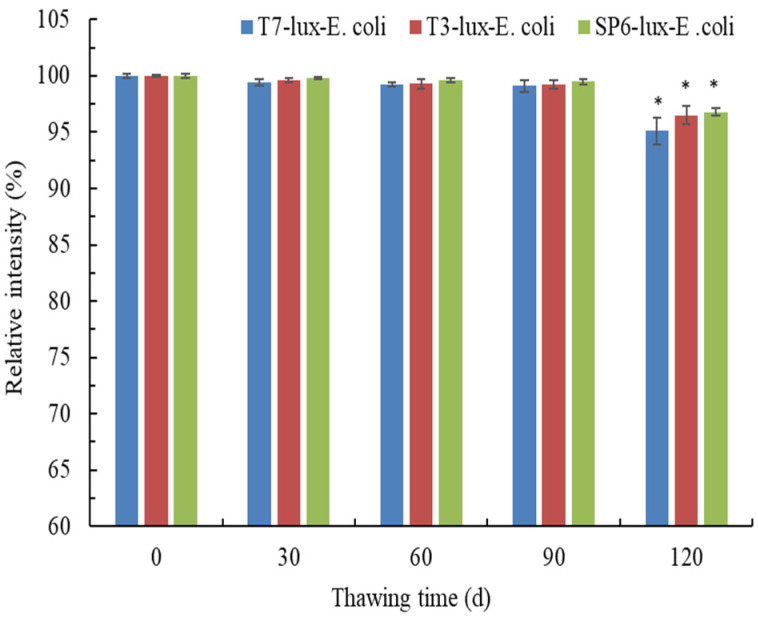
Effects of thawing time on luminescence intensities of three recombinant *E. coli* biosensors induced with 0.5 mg/L Cr(VI) for 1.5 h (T7-lux-*E. coli*), 1 h (T3-lux-*E. coli*), and 0.5 h (SP6-lux-*E. coli*). Data are expressed as the means ± standard deviations of three independent experiments (* *p* < 0.05 vs. blank control).

Regarding ease of use, constructing or preparing a recombinant biosensor when required is impractical. Thus, the luminescence intensities of the recombinant *E. coli* biosensors after cryogenic storage (30–120 days) were examined. The significant effects (*p* < 0.05) on the luminescence intensities of all thawing frozen cells after 120-day cryogenic storage were examined relative to fresh cells (0-day thawing time; Figure 5). After 90-day cryogenic storage, thawing recombinant T7-lux-*E. coli*, T3-lux-*E. coli*, and SP6-lux-*E. coli* cells maintained luminescence intensities of 99.1% ± 0.51%, 99.2% ± 0.38%, and 99.5% ± 0.25%, respectively. Similar results were found for the luminescence intensities of the three recombinant *E. coli* biosensors induced with 0.05 mg/L Cr(VI). Thus, the subsequent experiment used thawing frozen cells after 90-day cryogenic storage.

### 3.5. Relationship of Cr(VI) Concentration with Luminescence Intensity

**Figure 6:**
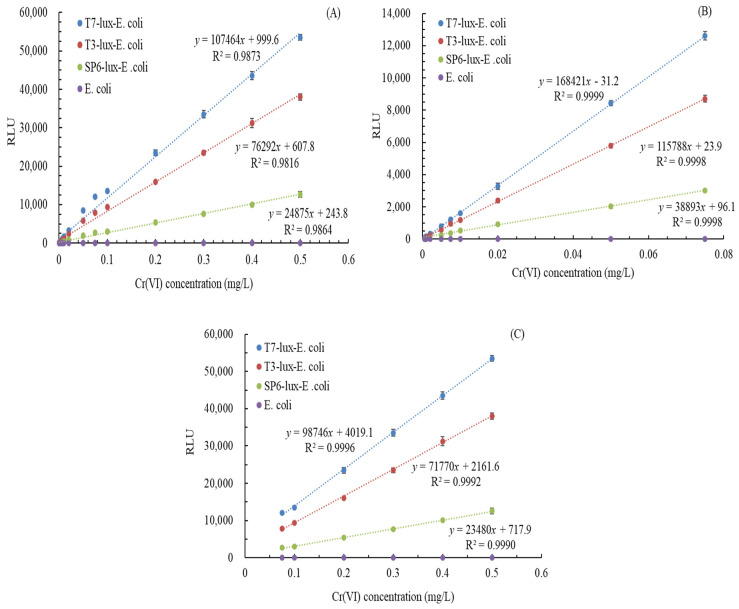
Relationship between Cr(VI) concentration [(**A**) 0–0.5 mg/L, (**B**) 0–0.075 mg/L and (**C**) 0.075–0.5 mg/L] and luminescence intensities of three recombinant *E. coli* biosensors (cell source: the thawing frozen cells after 90-day cryogenic storage; initial cell concentration: 5 × 10^6^ cfu/mL; culture media: modified TMM; operational condition: 37 °C and 200 rpm; incubation time: 1.5 h for T7-lux-*E. coli*, 1 h for T3-lux-*E. coli*, and 0.5 h for SP6-lux-*E. coli*.

According to the experimental results, we established the relationships between the Cr(VI) concentration and the luminescence intensity of the three recombinant *E. coli* biosensors using thawing frozen cells after 90-day cryogenic storage under optimal operating conditions. In this experiment, *E. coli* was used as a control. Figure 6A presents a set of regression equations for the Cr(VI) concentration and the luminescence intensity of the T7-lux-*E. coli*, T3-lux-*E. coli*, and SP6-lux-*E. coli* biosensors when the Cr(VI) concentration was 0–0.5 mg/L: *y* = 107,464*x* + 999.6, *y* = 76,292*x* + 607.8, and *y* = 24,875*x* + 243.8, respectively. Coefficients of determination (R^2^) of these regression equations indicated statistically significant results (>0.98), but the significance was insufficient for trace analysis of Cr(VI). Moreover, two obvious linear intervals between the Cr(VI) concentration and the luminescence intensity were noted. Thus, two other sets of relationships between the Cr(VI) concentration and luminescence intensity at various concentration ranges were further evaluated. Figure 6B presents another set of regression equations for the T7-lux-*E. coli*, T3-lux-*E. coli*, and SP6-lux-*E. coli* biosensors when the Cr(VI) concentration was ≤0.075 mg/L: *y* = 168,421*x* − 31.2, *y* = 115,788*x* + 23.9, and *y* = 38,893*x* + 96.1, respectively. R^2^ values of these regression equations were high (>0.999), indicating their reliability even if applied in trace analysis of Cr(VI). Figure 6C indicated another set regression equations for the T7-lux-*E. coli*, T3-lux-*E. coli*, and SP6-lux-*E. coli* biosensors while the Cr(VI) concentration ranged from 0.075 mg/L to 0.5 mg/L. These regression equations for these given data were *y* = 98,746*x* + 4019.1, *y* = 71,770*x* + 2161.6, and *y* = 23,480*x* + 717.9, respectively. R^2^ values for these equations were quite high (>0.999), representing their high reliability. The concentration-dependent differences in these linear relationships may be due to differences in promoter characteristics [9,40]. In addition, the LOD values of Cr(VI) measurement by the T7-lux-*E. coli*, T3-lux-*E. coli*, and SP6-lux-*E. coli* biosensors were calculated as 0.0005, 0.001, and 0.005 mg/L, respectively. Therefore, the T7*-*lux-*E. coli* biosensor was the most sensitive, and the SP6-lux-*E. coli* biosensor was the least sensitive. Table 2 summarizes LOD values and operating conditions of different biosensor for Cr(VI) measurement. Smutok et al. (2011) constructed a flavocytochrome *b2–*based *Hansenula polymorpha* recombinant biosensor, Branco et al. (2013) constructed a gfp-based recombinant *E. coli* biosensor, Bohrn et al. (2013) developed a V79 cell biosensor, Coelho et al. (2015) constructed a *gfp*-based recombinant *O. tritici* biosensor, Wang et al. (2016) developed an *Ochrobactrum anthropi* YC152 MFC-based biosensor, Wu et al. (2017) developed an *E. aestuarii* YC211 MFC-based biosensor, and Wu et al. (2019) developed a three-stage single-chambered MFC biosensor for Cr(VI) detection; their LODs for Cr(VI) were 0.52, 0.0194, 0.97, 0.388, 0.0125, 2.5, and 5 mg/L, respectively [5,6,10,13,16,17,31]. Compared with the aforementioned biosystems, our *luxAB*-based recombinant *E. coli* biosensors have smaller LODs (0.0005–0.005 mg/L), indicating their high sensitivity. However, it was inefficient in indicating the level of genotoxicity of Cr(VI) at low concentrations [36]. The satisfactory LOD of our sensors relative to previous sensors was due to the high activity of the *chrB* gene from *O. anthropi* YC211 and the position-appropriate promoters placed before the reporter gene *lux* [9].

Consequently, by virtue of the aforementioned reliable calibration curves for the Cr(VI) concentration range of 0.075–0.5 mg/L for the all biosensors or the Cr(VI) concentration range of 0.0005–0.75 mg/L (for T7-lux-*E. coli* biosensor), 0.001–0.75 mg/L (for T3-lux-*E. coli* biosensor), and 0.005–0.75 mg/L (for SP6-lux-*E. coli* biosensor), the Cr(VI) concentration in the water samples could be accurately and rapidly determined. In sum, the broad-range T7*-*lux-*E. coli* biosensor is a practical device for Cr(VI) measurement.

To evaluate the reproducibility of the biosensors for detecting Cr(VI), T7-lux-*E. coli*, T3-lux-*E. coli*, and SP6-lux-*E. coli* were tested under optimal conditions by using modified TMM with 0.5 mg/L Cr(VI). Relative standard deviation (RSD) for T7-lux-*E. coli*, T3-lux-*E. coli*, and SP6-lux-*E. coli* was 3.2, 3.6, and 3.3%, respectively (*n* = 10). The low values of RSD of our recombinant luminescent *E. coli* biosensors demonstrated operational stability.

### 3.6. Cr(VI) Detection in Real Water Samples by Using Our Three Recombinant Luminescent E. coli Biosensors

To date, some biosensors have been developed for detecting Cr(VI), but few biosensors have been applied for detecting the concentration of Cr(VI) in real water samples, particularly for determining compliance with the MAL in water bodies. Table 3 summarizes the measured Cr(VI) concentrations in three industrial effluent, three domestic effluent, and three surface water samples using the standard colorimetric method and our three recombinant luminescent *E. coli* biosensors. The results demonstrated that the Cr(VI) concentration determined using our biosensors and the colorimetric DPC method were highly correlated (R^2^ > 0.999), excluding those for the B water sample of surface water. The B water sample of surface water was difficult to measure using the colorimetric DPC method because the Cr(VI) concentration was lower than its LOD (0.01 mg/L). Moreover, the deviation between the Cr(VI) concentrations measured using the colorimetric DPC method and those measured using the T7-lux-*E. coli*, T3-lux-*E. coli*, and SP6-lux-*E. coli* biosensors was −5.0–7.9%, −11.0–14.6%, and −2.6–18.4%, respectively. Some water samples required dilution because the Cr(VI) concentration exceeded the linear measurement range (>0.5 mg/L). These samples (industrial effluents B and C and domestic effluents B and C) caused positive deviation when the T7-lux-*E. coli* biosensor was used, but it caused negative deviation when the T3-lux-*E. coli* and SP6-lux-*E. coli* biosensors were used. The SP6-lux-*E. coli* biosensor had high environmental adaptability, as mentioned previously; thus, it was accurate (−2.6–1.3%) when it was applied for Cr(VI) detection in complex matrices (e.g., industrial and domestic effluents). However, its performance was unfavorable when applied to detect low Cr(VI) concentrations (e.g., <0.03 mg/L), with high positive deviation (11.0–18.4%). By contrast, the performance of the T7-lux-*E. coli* biosensor was favorable when applied to detect low Cr(VI) concentrations (e.g., <0.03 mg/L), with low deviation (2.6–4.2%). Considering the measurement range and accuracy, the T7-lux-*E. coli* biosensor provided the most accurate and reliable Cr(VI) measurement in these aqueous matrices. However, for Cr(VI) detection in industrial and domestic effluents, the SP6-lux-*E. coli* biosensor was the optimal biosensor in terms of accuracy.

The measurement deviation of the T7-lux-*E. coli* biosensor for Cr(VI) detection in real water samples was much lower than that (−13–6.2%) of an *O. anthropi* YC152 MFC-based biosensor, and the measurement deviation of the T3-lux-*E. coli* and SP6-lux-*E. coli* biosensors was comparable to that of an *O. anthropi* YC152 MFC-based biosensor for Cr(VI) detection [6]. Taken together, these results clearly indicate that the developed recombinant luminescent bacterial biosensors can determine low Cr(VI) concentrations in different water bodies.

## 4. Conclusions

In this study, three novel recombinant *E. coli* biosensors containing the *chrB* gene (from *O. anthropi* YC211); the T3, T7, or SP6 promoter; and the reporter gene *lux* were constructed for the rapid and accurate measurement of low Cr(VI) concentrations in industrial effluent, domestic effluent, and surface water. Of these biosensors, the T7*-*lux-*E. coli* biosensor exhibited the highest illuminance intensity, the most sensitivity to Cr(VI) (LOD: 0.0005 mg/L), and the widest measurement range (0.0005–0.5 mg/L) for Cr(VI) concentrations. Moreover, the SP6-lux-*E. coli* biosensor had the shortest detection time (0.5 h) and the highest environmental adaptability but low LOD (0.005–0.5 mg/L). The three recombinant *E. coli* biosensors exhibited advantages over previously reported biosystems in terms of optimal LOD, wide measurement range, and low measurement deviation. To the best of our knowledge, this is the first report on the use of recombinant biosensors to monitor the Cr(VI) concentration in real wastewater, especially from the perspective of whether they meet water quality standards (e.g., Cr(VI) ≤ 0.05 mg/L). In sum, our biosensors, particularly the T7*-*lux-*E. coli* biosensor, are sensitive, reliable, specific, and stable systems for preliminary in-field detection of Cr(VI) in water samples.

## Figures and Tables

**Table 1:** Sequence of the primers used in this study.

Primer	Primer Sequence
Lux-Forward primer	
NdeI-T7-luxABf	CG*CA*↓*TATG*TAATACGACTCACTATAGGG**ATGAAGTTTGGAAATATTTG**
NdeI -T3-luxABf	CG*CA*↓*TATG*GCAATTAACCCTCACTAAAGG**ATGAAGTTTGGAAATATTTG**
NdeI -SP6-luxABf	CG*CA*↓*TATG*ATTTAGGTGACACTATAG**ATGAAGTTTGGAAATATTTG**
Lux-Reverse primer	
BamHI-luxABr	CG*G*↓*GATCC***TTAAGGCAGATTCTTTTC**
Chr-Forward primer	
XbaI-chrf	CG*T*↓*CTAGA***GATTGCTTATTCCTATTGCCA**
Chr-Reverse primer	
NdeI-chrr	CG*CA*↓*TATG***TCATACGCTGAGGGTCCCTTT**

....... indicates restriction enzymes recognition sequences; ↓ indicates restriction enzymes cutting sites; ___ indicates the T7, T3, or SP6 promoter sequence.

**Table 2:** LOD values and operating conditions of different biosensor for Cr(VI) measurement.

Sensor	LOD (mg/L)	Operating Conditions	References
*gfp*-based recombinant *O. tritici* biosensor	0.388	37 °C, pH 7, detection time: 5 h, batch	[5]
*O. anthropi* YC152 MFC-based biosensor	0.0125	35 °C, pH 7, detection time: 15 min, batch	[6]
*gfp*-based recombinant *E. coli* biosensor	0.0194	37 °C, pH 7, detection time: 5 h, batch	[10]
V79 cell biosensor	0.97	37 °C, pH 7, detection time: 3 h, batch	[13]
*E. aestuarii* YC211 MFC-based biosensor	2.5	30 °C, pH 7, detection time: 15 min, batch	[16]
three-stage single-chambered MFC biosensor	5	30 °C, pH 7, detection time: 6.6 min, continuous flow	[17]
flavocytochrome *b2–*based *H. polymorpha* recombinant biosensor	0.52	24 °C, pH 6.3, detection time: 20 min, batch	[31]
T7-biosensor	0.0005	37 °C, pH 7, detection time: 1.5 h, batch	This study
T3-biosensor	0.001	37 °C, pH 7, detection time: 1.0 h, batch	This study
SP6-biosensor	0.005	37 °C, pH 7, detection time: 0.5 h, batch	This study

**Table 3:** Cr(VI) measurement in real water samples by using the colorimetric DPC method and biosensors.

	Industrial Effluents			Domestic Effluents			Surface Water		
	A	B	C	A	B	C	A	B	C
DPC method	0.482 *	6.72	2.31	0.08	1.21	0.63	0.0212	ND **	0.0154
T7-biosensor	0.461 (−4.4%) ***	6.83 (1.6%)	2.42 (4.8%)	0.076 (−5.0%)	1.23 (1.7%)	0.68 (7.9%)	0.0221 (4.2%)	0.0061 (–)	0.0158 (2.6%)
T3-biosensor	0.491 (1.9%)	6.43 (−4.3%)	2.21 (−4.3%)	0.083 (3.8%)	1.18 (−2.5%)	0.59 (−6.3%)	0.0243 (14.6%)	0.0065 (–)	0.0137 (−11.0%)
SP6-biosensor	0.478 (−0.8%)	6.62 (−1.5%)	2.25 (−2.6%)	0.081 (1.3%)	1.19 (−1.7%)	0.62 (−1.6%)	0.0251 (18.4%)	0.0083 (–)	0.0171 (11.0%)

* Unit: mg/L; ** not determined; *** Deviation compared with the measured value using colorimetric DPC method.

## Data Availability

The data presented in this study are available on request from the corresponding author.
